# Neuroprotective Effects of Methyl 3,4-Dihydroxybenzoate against TBHP-Induced Oxidative Damage in SH-SY5Y Cells

**DOI:** 10.3390/molecules21081071

**Published:** 2016-08-22

**Authors:** Liang Cai, Li-Fang Wang, Jun-Ping Pan, Xiang-Nan Mi, Zheng Zhang, Hai-Ju Geng, Jia-Hui Wang, Song-Hui Hu, Wei Zhang, Qin Gao, Wu-Tian Wu, Huan-Min Luo

**Affiliations:** 1 Department of Pharmacology, School of Medicine, Jinan University, Guangzhou 510632, China; cai2011study@163.com (L.C.); wlifang001@163.com (L.-F.W.); panjunpingjnu319@163.com (J.-P.P.); Mrsmanjusaka@163.com (X.-N.M.); Jiapin@outlook.com (H.-J.G.); wjh8623@126.com (J.-H.W.); jinanyaolihu326@163.com (S.-H.H.); tgaoq@jnu.edu.cn (Q.G.); 2 School of International Education, Anhui Medical University, Hefei 230000, China; 3 School of Nursing, Guangdong Pharmaceutical University, Guangzhou 510632, China; 4 The First Affiliated Hospital of Jinan University, Guangzhou 510632, China; zhangzhengnc@163.com; 5 Department of Pathogen Biology and Medical Immunology, School of Basic Medicine, Ningxia Medical University, Yinchuan 750021, China; zhangweisj123@163.com; 6 Department of Anatomy, Li Ka Shing Faculty of Medicine of The University of Hong Kong, Hong Kong 999077, China; wtwu@hkucc.hku.hk; 7 Institute of Brain Sciences, Jinan University, Guangzhou 510632, China

**Keywords:** methyl 3,4-dihydroxybenzoate, oxidative stress, apoptosis, neuroprotection, nuclear factor erythroid 2-related factor 2

## Abstract

This study investigated the neuroprotective effects of methyl 3,4-dihydroxybenzoate (MDHB) against *t*-butyl hydroperoxide (TBHP) induced oxidative damage in SH-SY5Y (human neuroblastoma cells) and the underlying mechanisms. SH-SY5Y were cultured in DMEM + 10% FBS for 24 h and pretreated with different concentrations of MDHB or *N*-acetyl-l-cysteine (NAC) for 4 h prior to the addition of 40 μM TBHP for 24 h. Cell viability was analyzed using the methylthiazolyltetrazolium (MTT) and lactate dehydrogenase (LDH) assays. An annexin V-FITC assay was used to detect cell apoptosis rates. The 2′,7′-dichlorofluorescin diacetate (DCFH-DA) assay was used to determine intracellular ROS levels. The activities of antioxidative enzymes (GSH-Px and SOD) were measured using commercially available kits. The oxidative DNA damage marker 8-OHdG was detected using ELISA. Western blotting was used to determine the expression of Bcl-2, Bax, caspase 3, p-Akt and Akt proteins in treated SH-SY5Y cells. Our results showed that MDHB is an effective neuroprotective compound that can mitigate oxidative stress and inhibit apoptosis in SH-SY5Y cells.

## 1. Introduction

Oxidative stress is closely related to the pathogenesis of many diseases, including neurodegenerative disease [1]. Oxidative stress and cellular damage are found in a variety of sex- or age-related neurodegenerative diseases. These findings suggest that the administration of antioxidants and antioxidant inducers may be an effective clinical strategy for disease prevention and treatment [2].

All organisms have an antioxidant defense system to remove reactive oxygen species (ROS) and prevent the accumulation of ROS within a range of physiological conditions. However, the cell cannot remove excess ROS being accumulated beyond a certain threshold, which results in oxidative stress and induces many related-age diseases [3]. The study of oxidative stress reactions in nerve cells is largely focused on neurodegenerative diseases. Compared with the other organs, the brain is more susceptible to oxidative stress injury because the level of consumed oxygen is higher in the brain while the anti-oxidation ability is lower. Maintenance of the redox balance is crucial to normal brain functions. Oxidative stress likely plays a role in the pathogenesis of neurodegenerative diseases including Alzheimer’s disease (AD) [4], Parkinson’s disease (PD) [5], and Huntington’s disease (HD). Oxidation markers, such as lipid, protein, and nucleic acid oxidation products, are found in patients with these diseases [6]. Therefore, antioxidant supplements may prevent or at least delay the advancement of neurodegenerative diseases and reduce ROS-induced neuronal damage.

Cell apoptosis involves the characteristic cell shrinkage, nuclear pyknosis, DNA fragmentation and membrane vacuoles of autonomous, which activates a cysteine protease cascade of the caspase family and results in cell death [7]. ROS induces oxidative damage to DNA, which leads to apoptosis through an endogenous apoptotic pathway in mitochondria [8]. CytC, dATP and apoptotic protease-activating factor-1 (Apaf-1) compose the apoptosome [9], which activates caspase-9 and initiates the caspase cascade. Caspase-9 activates caspase-3, which is a key executor of apoptosis [10] to produce apoptosis-related morphological and biochemical change [11] The balance between the apoptotic-promoting Bax family proteins (e.g., Bad, Bax, and Bak) and the anti-apoptotic Bcl-2 family proteins (Bcl-XL and the Bcl-2) is a key factor. The ratio of Bcl-2/Bax is a “switch” that adjusts the mitochondrial permeability transition pore to activate or inhibit the cell death pathways of mitochondria [12].

The effect of bioactive small molecules extracted from plants on cell survival, apoptosis and the oxidative stress pathway has been widely studied in recent years. Methyl 3,4-dihydroxybenzoate (MDHB) is a small molecule compound that is extracted from East Asian Tang Materia, Malan and other natural plants [13]. Previous studies by our laboratory have demonstrated that MDHB has neurotrophic effects [14], anti-apoptotic effects induced by Aβ_25–35_ [15] and H_2_O_2_ [16] as well as the lifespan extension effects on *C. elegans* [17]. The present study used *t*-butyl hydroperoxide (TBHP) to induce cellular oxidative stress in cultured SH-SY5Y cells in vitro and examined the protective effect of MDHB on oxidative damaged in nerve cells and its associated mechanism. The results provide experimental evidence for oxidative stress-related neurodegenerative disease treatments for use in aging research.

## 2. Results 

### 2.1. The Effect of MDHB on SH-SY5Y Cell Viability

**Figure 1:**
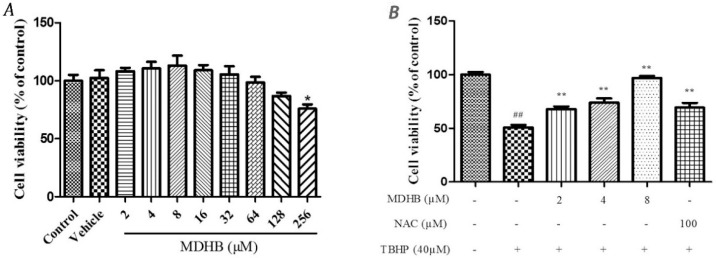

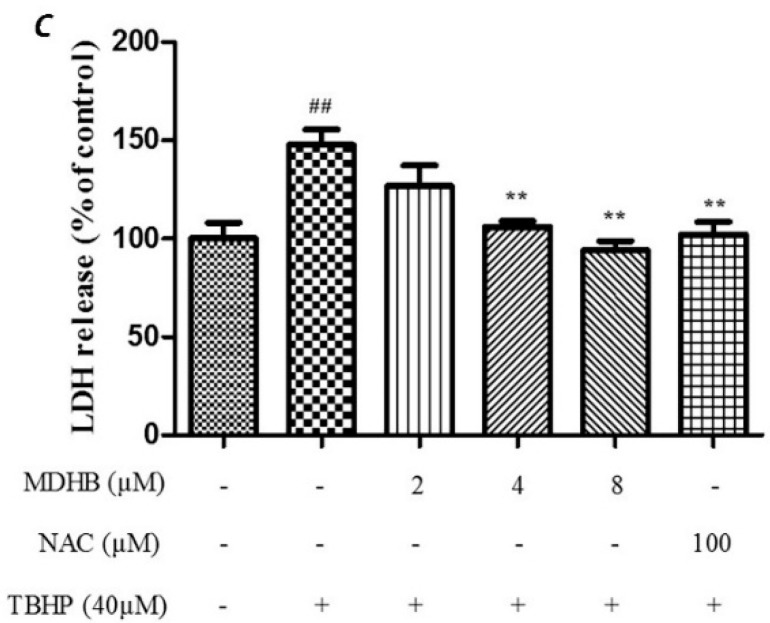
The effect of different concentration of MDHB on TBHP-treated SH-SY5Y cells by MTT assay and LDH assay. (**A**) The safety margin of MDHB. Cell viability was measured by MTT assay. SH-SY5Y cells were cultured with MDHB at 2~128 μM and 0.1% DMOS for 24 h. Data were normalized by control group. Values represent means ± S.E.M (*n* = 4); * *p* < 0.05 versus the TBHP-treated alone group (*n* = 4); (**B**) The cells were pretreated with 2, 4, or 8 μM of MDHB for 4 h, followed by treatment with 40 μM TBHP for 24 h. Cell viability was measured using the MTT assay. Data are normalized to the control group. Values represent means ± S.E.M. ^##^ *p <* 0.01 versus the control group. ** *p <* 0.01 versus the TBHP-treated alone group (*n* = 4); (**C**) Cells were pretreated with 2, 4, or 8 μM of MDHB for 4 h, followed by treatment with 40 μM TBHP for 24 h. LDH release was measured. Data are normalized to the control group. Values represent means ± S.E.M. ^##^ *p <* 0.01 versus the control group. ** *p <* 0.01 versus the TBHP-treated alone group (*n* = 4).

Cell viability was measured by MTT assay. First, The safety margin of MDHB, SH-SY5Y cells were cultured with MDHB at 2~128 μM and 0.1% DMOS for 24 h. The result showed that 0.1% DMSO and the concentration of 2~128 μM MDHB had no effect on the cell vitality (Figure 1A). SH-SY5Y cells were pretreated with 2, 4, or 8 μM of MDHB and 100 μM NAC for 4 h, followed by treatment with 40 μM TBHP for 24 h.

Cell viability was evaluated by MTT assay which showed that the cell viability of the TBHP model group was significantly lower than that of the control group (*p* < 0.01). The TBHP model group was compared with 2, 4, 8 µM MDHB and 100 μM NAC. Preprocessing group cell activity was significantly increased (*p* < 0.01) (Figure 1B). By detecting LDH release of cell-culture medium we can further analyze the cell activity effect of MDHB on the SH-SY5Y. The LDH assay detection result shows that LDH release of the TBHP model group was significantly increased compared with the control group (*p <* 0.01), the TBHP model group was compared with 2, 4, 8 µM MDHB and 100 μM NAC Preprocessing group LDH release was significantly increased (*p* < 0.01) (Figure 1C).

### 2.2. Effect of MDHB against TBHP-Induced Apoptosis in SH-SY5Y Cells

We used Annexin V/PI double-staining to detect the cell apoptosis rate using flow cytometry. The result shows that the cell apoptosis rate of the control group was 1.80% ± 0.59% and the cell apoptosis rate of the TBHP model group was obviously increased to 38.43% ± 3.09% (*p* < 0.01).

**Figure 2:**
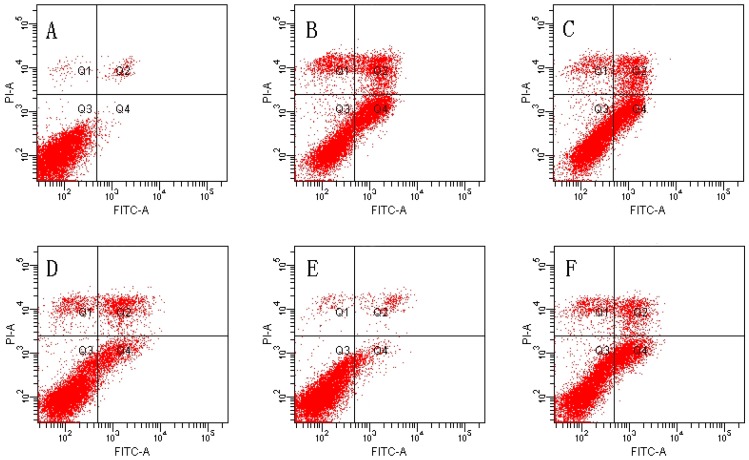

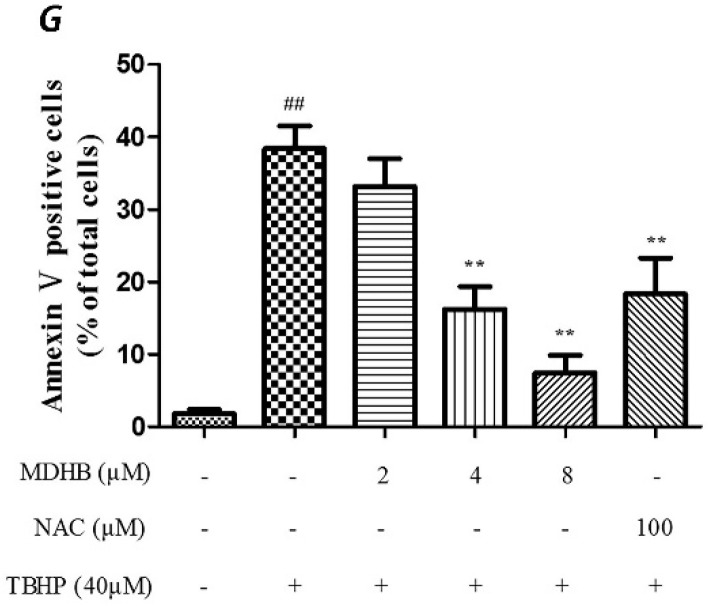
Effect of MDHB on TBHP-induced apoptosis in SH-SY5Y cells. (**A**) Control group; (**B**) 40 μM THBP group; (**C**) 2 μM MDHB + TBHP group; (**D**) 4 μM MDHB + TBHP group; (**E**) 8 μM MDHB + TBHP group; and (**F**) 100 μM NAC + TBHP group; The statistical result of annexin V/PI staining (**G**) showed that MDHB (2, 4, 8μM) can reduce RGC-5 cell apoptosis compared to H_2_O_2_-treated cells. Data are expressed as the mean ± S.E.M. ^##^ *p <* 0.01 versus the control group. ** *p <* 0.01 versus the TBHP-treated alone group (*n* = 3).

The TBHP model group was compared with 4, 8 µM MDHB and 100 μM NAC Preprocessing group the cell apoptosis rate was significantly decreased, only respectively for 16.23% ± 3.13%, and 7.47% ± 2.43%, and 18.33% ± 4.99% (*p* < 0.01) (Figure 2).

### 2.3. Effect of MDHB on DPPH Free Radical

DPPH is a stable radical with a single electron that exhibits strong absorption at 517 nm. The absorption gradually disappears when the radical scavenger is paired with its single-electron, and the alcoholic solution of DPPH radical becomes purple. Absorbance changes are directly proportional to the number of receiving electrons. The reagent indicates the scavenging ability of the DPPH radical.

**Figure 3:**
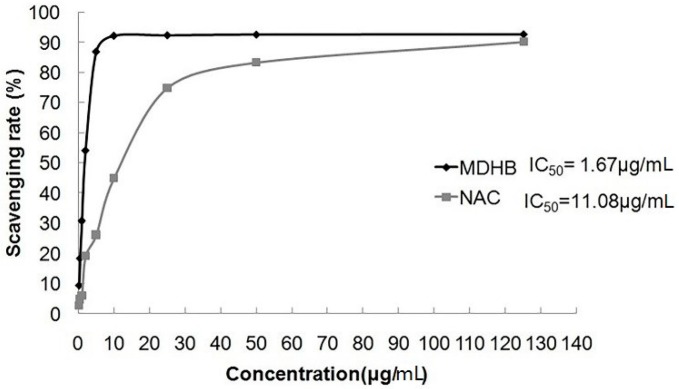
The scavenging rate of MDHB on DPPH free radical. Data are expressed as the mean ± S.E.M.

This experiment used different MDHB concentrations and the positive control drug NAC to investigate the radical scavenger rate of DPPH. MDHB exhibited strong scavenging ability for the DPPH radical (IC_50_ = 1.67 μg/mL), and the scavenging ability was stronger than NAC (IC_50_ = 11.08 μg/mL) (Figure 3).

### 2.4. Effect of MDHB on Intracellular ROS, SOD and GSH-Px Activity in SH-SY5Y Cells

**Figure 4:**
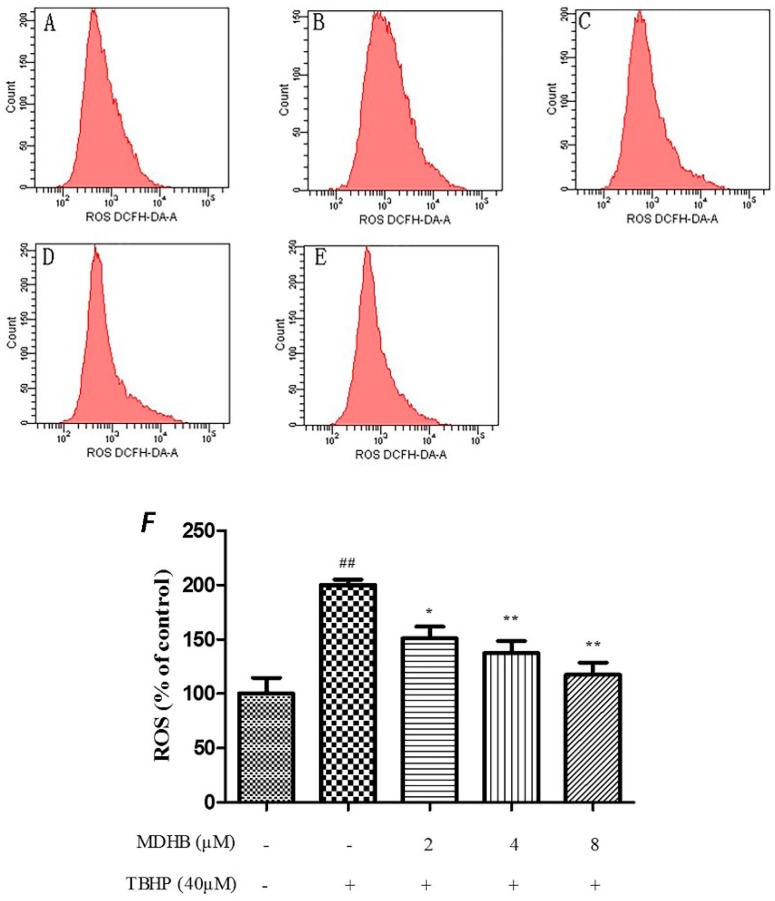
Effect of MDHB on TBHP-induced ROS accumulation in SH-SY5Y cells. SH-SY5Y cells were treated with MDHB (2, 4, or 8 μM) for 4 h before exposure to 40 μM TBHP for another 24 h. Intracellular ROS levels were measured via incubation with DCFH-DA fluorescent dye and analyzed using flow cytometry. (**A**) Control group; (**B**) 40 μM THBP group; (**C**) 2 μM MDHB + TBHP group; (**D**) 4 μM MDHB + TBHP group; and (**E**) 8 μM MDHB + TBHP group; The statistical result of DCFH-DA staining (**F**) showed that MDHB (2, 4, 8 μM) can reduce remarkably TBHP-induced ROS accumulation in SH-SY5Y cells. Values represent the mean ± S.E.M, ^##^ *p <* 0.01 versus the control group. * *p <* 0.05 versus the TBHP group. ** *p <* 0.01 versus the TBHP group (*n* = 3).

The level of ROS was analysed by flow cytometry by incubating SH-SY5Y cells with the fluorescent dye CM-H2DCFDA, The TBHP significantly increased intracellular ROS in SH-SY5Y cells relative to the control cells (*p* < 0.01), and that could be suppressed by MDHB (2, 4, 8 μM) (Figure 4).

**Figure 5:**
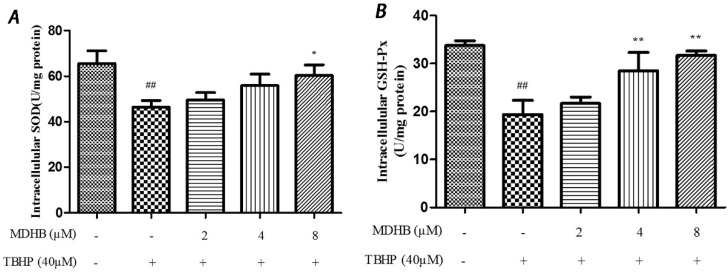

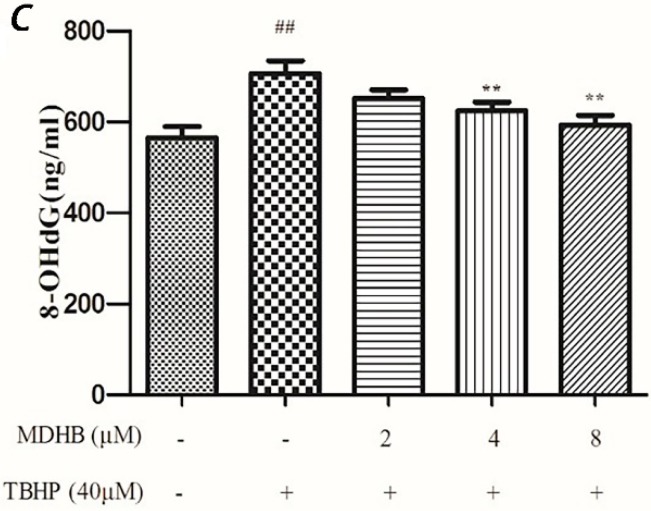
(**A**) Effect of MDHB on intracellular SOD activity in SH-SY5Y cells. SH-SY5Y cells were treated with MDHB (2, 4, or 8 μM) for 4 h before exposure to 40 μM TBHP for another 24 h. Intracellular SOD activity was estimated using WST-1 assay. Data are expressed as the mean ± S.E.M. ^##^ *p <* 0.01 versus the control group. * *p <* 0.05 versus the TBHP group (*n* = 4); (**B**) Effect of MDHB on intracellular GSH-Px activity in SH-SY5Y cells. Intracellular GSH-Px activity was estimated using a colorimetric assay. The data are expressed as the mean ± S.E.M. ^##^ *p <* 0.01 versus the control group, ** *p <* 0.01 versus the TBHP group (*n* = 3); (**C**) Effect of MDHB on 8-OHdG levels in the cultured media of SH-SY5Y cells. SH-SY5Y cells were treated with MDHB (2, 4, or 8 μM) for 4 h before exposure to 40 μM TBHP for another 24 h. 8-OHdG levels in the cultured media of SH-SY5Y cells were detected using ELISA. The data are expressed as the mean ± S.EM. ^##^ *p <* 0.01 versus control group. ** *p <* 0.01 versus TBHP group (*n* = 3).

SOD is an important antioxidant enzyme that scavenges radical and protects cells from damage. GSH-Px is specifically catalyzed by reductive glutathione GSH to the reduction reaction of H_2_O_2_ to catalyze H_2_O_2_ decomposition and protect cells. This experiment used the SOD and GSH-Px kits to detect intracellular SOD and GSH-Px vitality, which reflected anti-oxidative stress ability. The results are shown in (Figure 5A,B).

### 2.5. Effect of MDHB on 8-OHdG Levels in the Cultured Media of SH-SY5Y Cells

8-OHdG is a specific product of DNA oxidative damage, and it can be used as a marker to evaluate DNA oxidative damage. We adopted the ELISA test to determine 8-OHdG content in cell culture supernatants to reflect the degree of DNA oxidative damage in cells. The results show that MDHB reduced the content of 8-OHdG in cell culture supernatants, which supports that MDHB reduced oxidative stress-induced DNA damage in SH-SY5Y cells (Figure 5C).

### 2.6. Effect of MDHB on Protein Expression of the Related Protein

**Figure 6:**
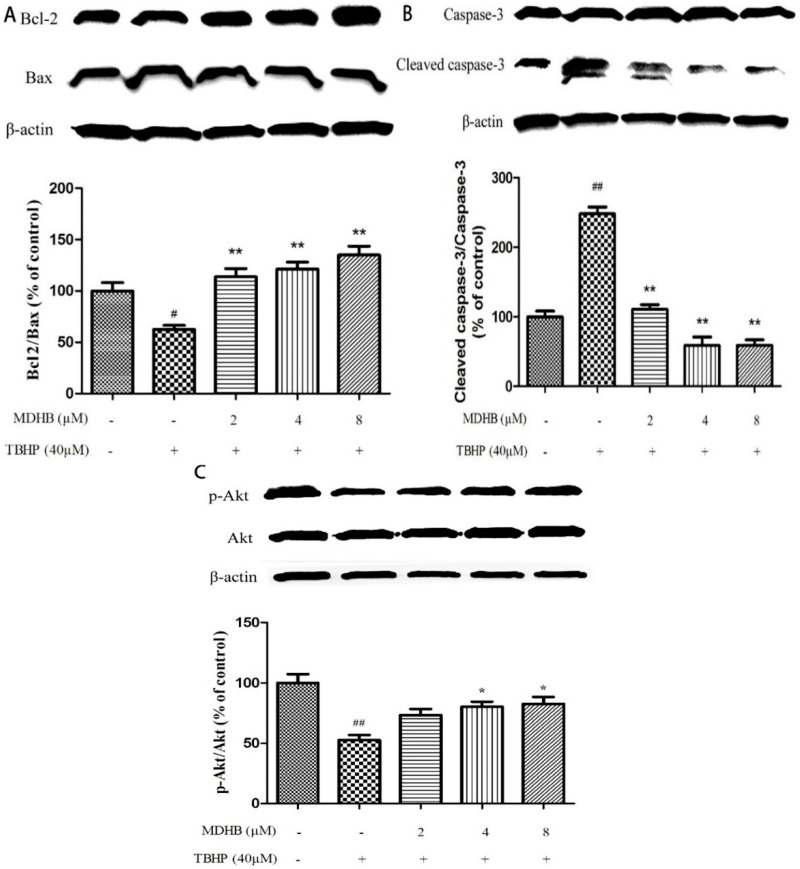
Effect of MDHB on Bcl2/Bax, cleaved caspase-3/pro-caspase-3, p-Akt/Akt expression in SH-SY5Y cells. Protein expression was estimated using Western blot. (**A**) Effect of MDHB on Bcl2/Bax expression in SH-SY5Y cells. The data are expressed as the mean ± S.EM. ^#^ *p <* 0.05 versus the control group. ** *p <* 0.01 versus the TBHP group (*n* = 3); (**B**) Effect of MDHB on the expression of cleaved caspase-3/pro-caspase-3 in SH-SY5Y cells. The data are expressed as the mean ± S.EM. ^##^ *p <* 0.01 versus the control group. ** *p* *<* 0.01 versus the TBHP group (*n* = 3); (**C**) Effect of MDHB on p-Akt/Akt expression in SH-SY5Y cells. The data are expressed as the mean ± S.EM. ^##^ *p <* 0.01 versus control group, * *p <* 0.05 versus TBHP group (*n* = 3).

To further explore the molecular mechanisms of neuroprotective effect of MDHB on THBP-induced oxidative damage, we examined the expression of Bcl2, Bax, caspase-3, p-Akt and Akt in SH-SY5Y cells by western blot. The results showed that treatment of cells with TBHP alone caused a significant decrease in Bcl-2/Bax level as compared with control group. However, pretreatment of cells with MDHB (2, 4, 8 μM) could up-regulate Bcl-2/Bax level (Figure 6A). In addition, TBHP could also increase the level of cleaved caspase-3, while MDHB at concentrations of 2, 4, 8 μM could reverse the trend (Figure 6B), which means that MDHB could significantly prevented the activation of caspase-3 induced by TBHP. MDHB could increase activation of Akt kinases as determined by the phosphorylation of Akt (Figure 6C). These results suggested that the neuroprotective effect of MDHB on TBHP-induced neurotoxicity may be, or at least in part, mediated by regulating the expression of apoptosis-related proteins Bcl-2, Bax, caspase-3 and activation of Akt kinases.

## 3. Discussion

Oxidative stress is the result of an imbalance between ROS production and the antioxidant defense. ROS oxidizes the vital components of cells, such as lipids, proteins and nucleic acids, which may induce cell damage and apoptosis or necrosis [18]. Oxidative stress is the primary cause of neurodegenerative diseases [19,20]. TBHP is an ROS inducer that can stimulate the excessive production of ROS, produce malondialdehyde, reduce GSH, and induce cell apoptosis [21,22,23]. SH SY5Y cells treated with 40 μM TBHP for 24 h was established as a model of oxidative stress damage in this experiment.

MDHB is a highly fat-soluble small molecule compound that exists in a variety of natural plants, previously study has confirmed that MDHB exerts a neurotrophic effect on primary neurons [14]. The safety range of MDHB in SH-SY5Y cells was explored by MTT and used to establish the effective concentration of MDHB in the TBHP-induced oxidative damage model. NAC is a common antioxidant that can directly react with ROS, promote the synthesis of GSH [24,25] and inhibit the apoptosis of nerve cells [26], in this experiment 100 μM NAC was used as a positive control. The MTT results demonstrated that 2, 4, and 8 μM MDHB and 100 μM NAC pretreatment obviously increased the activity of oxidative damage in SH-SY5Y cells. The release of LDH reflects the loss of cell membrane integrity. Therefore, extracellular LDH activity may be a simple and reliable index of cell death [27]. The LDH results were confirmed because 2, 4, and 8 μM MDHB improved the activity of oxidative damage in SH-SY5Y cells.

ROS is a critical factor in the brain aging process, and it interferes with mitochondrial respiration. ROS increases with age, and brain function is altered. The mitochondrial electron transport chain complex I and complex III leak out electrons combined with O_2_ to generate ROS, which is the source of ROS in the brain. Complex I releases superoxide (O_2_^−^) to the substrate, and complex III releases superoxide (O_2_^−^) to the mitochondrial membrane. Mitochondrial dysfunction induces abundant release of ROS, and weakens the activity of antioxidant enzyme (for example, in the elderly). An excess of free radicals and imbalance in calcium homeostasis was observed in AD patients [28]. We adopted the DCFH-DA method to detect the concentration of intracellular reactive oxygen, and the results demonstrated that 2, 4, and 8 μM MDHB significantly reduced intracellular ROS.

α,α-Diphenyl-β-picrylhydrazyl (DPPH) is a stable radical with a nitrogen center that has been widely used to evaluate the performance of antioxidant-scavenging free radicals [29]. The DPPH free radical alcohol solution is purple, and its absorption gradually disappears when a free radical scavenger is paired with its single electron. The extent of the reaction mixture’s color change reflects the concentration and titer of antioxidants. A lower absorbance demonstrates a higher activity of DPPH radical scavenging [30]. We adopted a chemical simulation to test the impact of MDHB on DPPH’s radical-scavenging ability, and the results demonstrated that MDHB was a strong scavenger of DPPH radicals (IC_50_ = 1.67 μg/mL).

ROS-induced DNA peroxidation generates gene mutations that affect the stability of microtubules and combine with transcription factors to block gene transcription. The primary products of DNA oxidative damage include 8-hydroxyadenine, 8-OH-Ade, 8-hydroxyguanine, 8-OH-Gua and its deoxyuridine equivalent 8-hydroxydeoxyguanosine (8-OHdG) and thymine glycol [31]. 8-OHdG is the specific product of DNA oxidative damage that may be used as a marker of DNA oxidative damage. We used ELISA to determine the content of 8-OHdG in cell culture supernatants, which reflected the degree of DNA oxidative damage in cells. The results demonstrated that MDHB reduced the content of 8-OHdG in cell culture supernatants, which suggests that MDHB reduced oxidative stress-induced DNA damage in SH-SY5Y cells.

A system of enzymes and non-enzymes maintain the balance of active oxygen in the body to limit the accumulation of ROS. Enzymes of the antioxidant system include SOD, GSH-Px, thioredoxin reductase (TR) and catalase (CAT) [32]. SOD is an important antioxidase in the body that converts O_2_^•−^ into O_2_ and H_2_O_2_. GSH-Px specifically catalyzes the reduction reaction of reduced glutathione GSH to H_2_O_2_ to catalyze the decomposition of H_2_O_2_ and protect cells. We examined intracellular SOD and the ability of GSH-Px to reflect anti-oxidative stress in each group in this study. The results demonstrate that MDHB improved anti-oxidase SOD and GSH-Px in vivo, which indicates that MDHB improved the defense of the cells to oxidative stress. Oxidative stress damage induces cell apoptosis. This experiment used Annexin V/PI staining and flow cytometry to detect the apoptosis rate of SH-SY5Y cells. The results demonstrated that TBHP induced cell apoptosis, and MDHB suppressed the apoptosis of TBHP-induced apoptosis. Because these pathways of oxidative stress, cell apoptosis and antioxidant defense intersect, oxidative damage resulting in cell apoptosis and survival depends on the degree of cell damage and the cells’ reaction [33].

The biochemical changes in the early stages of apoptosis induce mitochondrial dysfunction, including the opening of cell membrane channels, the release of cytochrome C, the protein expression changes of the Bcl-2 family and the activation of caspases [34,35]. The Bcl-2 family proteins are key components of the cascade reaction of mediated ectophragm permeability apoptosis [36]. The balance between the promotion of apoptotic Bax family proteins (Bad, Bax, and Bak) and anti-apoptotic Bcl-2 family proteins (Bcl-XL and the Bcl-2) is a key factor in cell apoptosis. The ratio of Bcl-2/Bax acts as a “switch” by adjusting the mitochondrial permeability transition pore to activate or inhibit the mitochondrial apoptosis [12], Literature reports indicate that activating the Bcl-2 family proteins (such as the Bcl-2, Bak1 and Bax) and caspase-9 encoding gene induces cell apoptosis. Caspase-9 activates caspase-3, which is a key component of the exogenous cell death receptor pathway and endogenous mitochondrial apoptosis pathway [10]. Activated caspase-3 catalyzes proteins in cells, such as the degradation of PARP. PARP is an important ribozyme in DNA repair, cell proliferation, apoptosis and transcription, and it is abundant in cells [37]. However, activated caspase-3 can shear 116-kDa PARP into 89 kDa segments of apoptosis, which induces the lack of DNA repair and DNA damage and apoptosis [38]. In this experiment we found that 40 μM TBHP significantly reduced the ratio of Bcl-2/Bax in SH-SY5Y cells at 24 h and activated the expression of caspase-3. However, the ratio of Bcl-2/Bax notably increased after the addition of MDHB, and the activation of caspase-3 was obviously inhibited. The results demonstrated that MDHB could protect SH-SY5Y cells from TBHP-induced oxidative damage possibly by inhibiting ROS accumulation, reducing DNA oxidative damage, activating PI3K/Akt pathway and modulating the expression of apoptosis-related proteins. Akt is a serine/threonine kinase. It can be activated by phosphorylation and subsequently activates multiple downstream targets to enhance cell survival. Akt could promote cell survival by its abilities to phosphorylate Bad at Ser136; Akt also directly inhibits activation of caspase-9 by phosphorylating pro-caspase-9 at Ser-196 and by this inhibits proteolytic processing of pro-Caspase-9, Caspase-9 activates caspase-3, which is a key component of the exogenous cell death receptor pathway and endogenous mitochondrial apoptosis pathway, so phosphorylated Akt can have a neuroprotective effect against TBHP-induced oxidative damage in SH-SY5Y cells).

## 4. Materials and Methods 

### 4.1. Materials 

SH-SY5Y cells were purchased from the Institute of Basic Medical Sciences of Chinese Academy of Medical Sciences (Guangzhou, Guangdong, China). High-glucose Dulbecco’s modified Eagle’s medium (DMEM), fetal calf serum (FBS) and trypsin-EDTA solution were purchased from Gibco (Grand Island, NY, USA). Cell culture plates were purchased from Jet Biofil (Guangzhou, Guangdong, China). MDHB (No. L09552) was obtained from the Alfa Aesar Co. (Ward Hill, MA, USA). MTT, DMSO and DPPH-free radicals were purchased from Sigma (St. Louis, MO, USA). TBHP (No. B106035) was purchased from Aladdin (Shanghai, China). Malonyldialdehyde (MDA), LDH, GSH-Px and superoxide dismutase (SOD) assay kits were obtained from Nanjing Jiancheng Bioengineering Institute (Nanjing, Jiangsu, China). The Apoptosis Detection Kit, Reactive Oxygen Species Assay Kit and BCA Protein Assay Kit were obtained from the BiYunTian Biotech Company (Shanghai, China). The human 8-OHdG ELISA kit was obtained from Cusabio (Barksdale, DE, USA). Antibodies against Akt, phospho-Akt and Bcl-2, Bax, caspase-9, and caspase-3 were purchased from Cell Signaling Technology (Boston, MA, USA).

### 4.2. Culture of SH-SY5Y Cells

SH-SY5Y cells were maintained in DMEM containing 10% FBS in a humidified atmosphere of 95% air and 5% CO_2_ at 37 °C. SH-SY5Y cells were passaged using 0.25% trypsinization, seeded into 96-well plates (4.0 × 10^3^ cells/cm^2^) and cultured for 24 h. Cells were pretreated with different concentrations of MDHB (2, 4, 8, 16, and 32 mM) dissolved in DMSO (Sigma) and cultured for 24 h in different concentrations of TBHP (10, 20, 40, 60, and 80 mM). Cells in this experiment were divided into six groups: the control group, vehicle + 40 μM THBP group, 2 μM MDHB + TBHP group, 4 μM MDHB + TBHP group, 8 μM MDHB + TBHP group, and 100 μM NAC + TBHP group.

### 4.3. MTT Assay

Cell viability was evaluated using the MTT assay. SH-SY5Y cells were cultured in a 96-well culture plate at a density of 4.0 × 10^3^ cells/cm^2^ for 24 h, and the supernatant was discarded. Each group was pretreated with different reagents for 4 h. Reagents and 40 μM TBHP were added, 24 h later the media were removed. DMEM (100 μL) with 10% MTT was added to each well, and kept at 37 °C, 4 h later the media was discarded. DMSO was added and kept away from light on a shaker for 10 min. Absorption was measured at 570 nm using a Bio-Rad 400 microplate reader (Bio-Rad, Hercules, CA, USA). Experiments were repeated at least 3 times and compared with control experiments. Results were compared using one-way analysis of variance followed by Dunnett’s t-test. 

### 4.4. LDH Assay

SH-SY5Y cells were cultured in a 96-well culture plate at a density of 4.0 × 10^3^ cells/cm^2^ for 24 h, and the supernatants were discarded. Each group consisted of a set of six wells. Reagents and 40 μM TBHP were added after 4 h, and the media were removed after 24 h. The media were centrifuged at 1000 r/min for 1 min, and the supernatants were collected to be used in LDH assay, which was performed according to the manufacturer’s instructions. 

### 4.5. Detection of Cell Apoptosis by Flow Cytometry 

Cell apoptosis was detected using the Annexin V-FITC/PI apoptosis detection kit. SH-SY5Y cells were seeded into 6-well culture plates (4.0 × 10^3^ cells/cm^2^) and cultured for 24 h. The medium was removed after these groups were treated, and the cells were rinsed once with 0.1 M PBS. Cells were passaged using 0.25% trypsinization for 2 min and collected in centrifuge tubes. Cells were centrifuged at 1500 r/min for 5 min, and supernatants were discarded. Binding Buffer (200 μL) was added to each tube and blended. Annexin V-FITC (5 μL) was added for 10 min at room temperature in the dark, and tubes were centrifuged at 1000 r/min for 5 min. Supernatants were discarded, and 200 μL Binding Buffer was added to resuspend the cells. PI (5 μL) was added blended for testing.

### 4.6. Chemical Simulation System to Detect the Scavenging Ability of MDHB on DPPH Free Radicals

0.2 mM DPPH, 1.0 mg/mL MDHB and 1.0 mg/mL NAC stock solutions were prepared with anhydrous ethanol. Different testing concentrations (0.25, 0.5, 1, 2, 5, 10, 25, 50, and 125 μg/mL) were diluted in anhydrous ethanol, A sample solution (1 mL) was mixed well with 1 mL DPPH solution at room temperature for 30 min, and the absorption was measured at 517 nm using a Bio-Rad 400 microplate reader (Bio-Rad, Hercules, CA, USA). The absorptions of 1 mL anhydrous ethanol 1 mL DPPH solution and a 1 mL anhydrous ethanol and 1 mL sample solution were compared.

### 4.7. Detection of Cell Reactive Oxygen Species Using the DCFH-DA Assay by Flow Cytometry

SH-SY5Y cells were seeded into 6-well culture plates (4.0 × 10^3^ cells/cm^2^) and cultured for 24 h. The media was removed after these groups were treated. Cells were rinsed once with 0.1 M PBS and passaged using 0.25% trypsinization for 2 min. Cells were collected in centrifuge tubes and centrifuged at 1500 r/min for 5 min, and the supernatants were discarded. DCFH-DA (200 μL) was added to each tube for 20 min at 37 °C in the absence of light. A keratinocyte serum-free medium (500 μL) was added to rinse the cells, and cells were centrifuged at 1000 r/min for 5 min. The supernatants were discarded, and 200 μL keratinocyte serum-free medium was added for testing.

### 4.8. Determination of Intracellular SOD Activity

SH-SY5Y cells were seeded in 6-well culture plates (4.0 × 10^3^ cells/cm^2^) and cultured for 24 h. The media were removed after these groups were treated. Cells were rinsed once with 0.1 M PBS and passaged using 0.25% trypsinization for 2 min. Cells were collected in centrifuge tubes and centrifuged at 1500 r/min for 5 min, and supernatants were discarded. PBS (0.1 M) was added to the supernatant of each tube. All of the samples were placed in an ice-water bath, and cells were lysed using ultrasound (300 W ultrasonic power, once every 5 s, 4 times). A portion of the samples was retained to detect protein concentrations by BCA assay. The remaining portion of the samples was processed according to the SOD kit instructions.

### 4.9. Detection of 8-Hydroxydeoxyguanosine by ELISA

SH-SY5Y cells were seeded into 6-well culture plates (4.0 × 10^3^ cells/cm^2^). The media were removed after the groups were treated. Cells were centrifuged at 3000 r/min for 15 min, and the supernatants were removed. Each reagent was prepared 30 min before the experiments at room temperature, and the wash buffer was also mixed. We set an empty control well and standard wells that contained 50 μL biological reference preparations. Two biological reference wells were set, and each well contained 50 μL of test sample. The enzyme substrate complex (50 μL) was added to each well, except the control well and blended at 37 °C for 1 h. Plates were washed 3 times and patted to dry. TMB Substrate A (50 μL) and TMB Substrate B (50 μL) were blended in each well at 37 °C for 15 min in the absence of light. Stop buffer (50 μL) was added to each well to stop the reaction, and the absorption was measured at 450 nm using the Bio-Rad 400 microplate reader.

### 4.10. Detection of Related Proteins Using Western Blotting

The expression levels of Bax, Bcl-2, caspase-3, p-akt and akt proteins were determined using western blotting. SH-SY5Y cells were plated in 6-well plates at a density of 9.5 × 10^6^ cells per well and treated as described above. Cells were incubated with MDHB and TBHP, and the medium was removed. SH-SY5Y cells were lysed in cell lysis buffer at 4 °C for 40 min, and the lysate was centrifuged at 10,000 r/min for 5 min at 4 °C. Equal amounts of protein were separated on 12% SDS-PAGE gels and transferred to polyvinylidene fluoride (PVDF) membranes. Membranes were blocked with 5% (*v*/*w*) skimmed milk powder in Tris-buffered saline with 0.1% Tween 20 (TBST) for 1 h at room temperature. Membranes were washed three times with TBST and incubated with different primary antibodies (anti-Bcl-2, anti-Bax, anti-caspase-3, anti-akt, anti-p-akt and anti-β-actin) overnight at 4 °C. Membranes were washed three times with TBST the following day and incubated with a second antibody (anti-rabbit IgG) at room temperature for 1 h. Membranes were washed three times with TBST, and protein immuno-complexes were visualized using BeyoECL Plus. Blots were scanned and quantified with Quantity One software (4.62, Bio-Rad, Hercules, CA, USA). 

### 4.11. Statistical Analyses 

All data are expressed as the mean ± S.E.M. Statistical significance was assessed by using a *t* test or ANOVA followed by Boferonni’s test, using the Prism software (GraphPad, San Diego, CA, USA). A value of *p* < 0.05 was considered as significantly different from the control.

## Abbreviations

The following abbreviations are used in this manuscript:

MDHB: Methyl 3,4-dihydroxybenzoate
TBHP: t-Butylhydroperoxide
NAC: *N*-Acetyl-l-cysteine
ROS: Reactive oxygen species
LDH: Lactic dehydrogenase
SOD: Superoxide dismutase
GSH-Px: Glutathione peroxidase
8-OhdG: 8-Hydroxy-2-deoxyguanosine
DPPH: α,α-Diphenyl-β-picrylhydrazyl
Nrf2: Nuclear factor E2-related factor 2

## References

[1] Jomova K., Vondrakova D., Lawson M., Valko M. (2010). Metals, oxidative stress and neurodegenerative disorders. Mol. Cell. Biochem..

[2] Barone E., Cenini G., di Domenico F., Martin S., Sultana R., Mancuso C., Murphy M.P., Head E., Butterfield D.A. (2011). Long-term high-dose atorvastatin decreases brain oxidative and nitrosative stress in a preclinical model of Alzheimer disease: A novel mechanism of action. Pharmacol. Res..

[3] Martindale J.L., Holbrook N.J. (2002). Cellular response to oxidative stress: Signaling for suicide and survival. J. Cell. Physiol..

[4] Von Arnim C.A., Gola U., Biesalski H.K. (2010). More than the sum of its parts? Nutrition in Alzheimer’s disease. Nutrition.

[5] Mandel S., Grunblatt E., Riederer P., Gerlach M., Levites Y., Youdim M.B. (2003). Neuroprotective strategies in Parkinson’s disease: An update on progress. CNS Drugs.

[6] Seet R.C., Lee C.Y., Lim E.C., Tan J.J., Quek A.M., Chong W.L., Looi W.F., Huang S.H., Wang H., Chan Y.H. (2010). Oxidative damage in Parkinson disease: Measurement using accurate biomarkers. Free Radic. Biol. Med..

[7] Budihardjo I., Oliver H., Lutter M., Luo X., Wang X. (1999). Biochemical pathways of caspase activation during apoptosis. Annu. Rev. Cell. Dev. Biol..

[8] Kubli D.A., Gustafsson A.B. (2012). Mitochondria and mitophagy: The yin and yang of cell death control. Circ. Res..

[9] Caroppi P., Sinibaldi F., Fiorucci L., Santucci R. (2009). Apoptosis and human diseases: mitochondrion damage and lethal role of released cytochrome C as proapoptotic protein. Curr. Med. Chem..

[10] Garcimartin A., Merino J.J., Gonzalez M.P., Sanchez-Reus M.I., Sanchez-Muniz F.J., Bastida S., Benedi J. (2014). Organic silicon protects human neuroblastoma SH-SY5Y cells against hydrogen peroxide effects. BMC Complement. Altern. Med..

[11] Mazumder S., Plesca D., Almasan A. (2008). Caspase-3 activation is a critical determinant of genotoxic stress-induced apoptosis. Methods Mol. Biol..

[12] Wang N., Feng Y., Zhu M., Tsang C.M., Man K., Tong Y., Tsao S.W. (2010). Berberine induces autophagic cell death and mitochondrial apoptosis in liver cancer cells: The cellular mechanism. J. Cell. Biochem..

[13] Xu W., Gong X., Zhou X., Zhao C., Chen H. (2010). Chemical constituents and bioactivity of *Kalimeris indica*. Zhongguo Zhong Yao Za Zhi.

[14] Zhang Z., Zhou X., Zhou X., Xu X., Liao M., Yan L., Lv R., Luo H. (2012). Methyl 3,4-dihydroxybenzoate promotes neurite outgrowth of cortical neurons cultured in vitro. Neural Regen. Res..

[15] Zhou X.W., Zhang Z., Su C.F., Lv R.H., Zhou X., Cai L., Wang C.Y., Yan L., Zhang W., Luo H.M. (2013). Methyl 3,4-dihydroxybenzoate protects primary cortical neurons against Aβ_25–35_-induced neurotoxicity through mitochondria pathway. J. Neurosci. Res..

[16] Zhou X., Su C.F., Zhang Z., Wang C.Y., Luo J.Q., Zhou X.W., Cai L., Yan L., Zhang W., Luo H.M. (2014). Neuroprotective effects of methyl 3,4-dihydroxybenzoate against H_2_O_2_-induced apoptosis in RGC-5 cells. J. Pharmacol. Sci..

[17] Zhang W., Cai L., Geng H.J., Su C.F., Yan L., Wang J.H., Gao Q., Luo H.M. (2014). Methyl 3,4-Dihydroxybenzoate Extends the Lifespan of *Caenorhabditis elegans*, Partly via W06A7.4 Gene. Exp. Gerontol..

[18] Abarikwu S.O., Pant A.B., Farombi E.O. (2012). 4-Hydroxynonenal induces mitochondrial-mediated apoptosis and oxidative stress in SH-SY5Y human neuronal cells. Basic Clin. Pharmacol. Toxicol..

[19] Gilgun-Sherki Y., Melamed E., Offen D. (2001). Oxidative stress induced-neurodegenerative diseases: The need for antioxidants that penetrate the blood brain barrier. Neuropharmacology.

[20] Tezel G. (2006). Oxidative stress in glaucomatous neurodegeneration: Mechanisms and consequences. Prog. Retin. Eye Res..

[21] Bhattacharya S., Gachhui R., Sil P.C. (2011). Hepatoprotective properties of kombucha tea against TBHP-induced oxidative stress via suppression of mitochondria dependent apoptosis. Pathophysiology.

[22] Amoroso S., D′Alessio A., Sirabella R., di Renzo G., Annunziato L. (2002). Ca^2+^-independent caspase-3 but not Ca(2+)-dependent caspase-2 activation induced by oxidative stress leads to SH-SY5Y human neuroblastoma cell apoptosis. J. Neurosci. Res..

[23] Kanupriya, Prasad D., Sai Ram M., Sawhney R.C., Ilavazhagan G., Banerjee P.K. (2007). Mechanism of *tert-*butylhydroperoxide induced cytotoxicity in U-937 macrophages by alteration of mitochondrial function and generation of ROS. Toxicol. In Vitro.

[24] Gurer H., Ercal N. (2000). Can antioxidants be beneficial in the treatment of lead poisoning?. Free Radic. Biol. Med..

[25] Da Silva D.G., Ricci O., de Almeida E.A., Bonini-Domingos C.R. (2015). Potential utility of melatonin as an antioxidant therapy in the management of sickle cell anemia. J. Pineal. Res..

[26] Rushworth G.F., Megson I.L. (2014). Existing and potential therapeutic uses for *N*-acetylcysteine: The need for conversion to intracellular glutathione for antioxidant benefits. Pharmacol. Ther..

[27] Cheng Y., Zhang L., Sun W., Tang J., Lv Z., Xu Z., Yu H. (2014). Protective effects of a wheat germ peptide (RVF) against H_2_O_2_-induced oxidative stress in human neuroblastoma cells. Biotechnol. Lett..

[28] Hashimoto M., Rockenstein E., Crews L., Masliah E. (2003). Role of protein aggregation in mitochondrial dysfunction and neurodegeneration in Alzheimer′s and Parkinson′s diseases. Neuromol. Med..

[29] Wang X., Wu Q., Wu Y., Chen G., Yue W., Liang Q. (2012). Response surface optimized ultrasonic-assisted extraction of flavonoids from *Sparganii rhizoma* and evaluation of their in vitro antioxidant activities. Molecules.

[30] Akanni O.O., Owumi S.E., Adaramoye O.A. (2014). In vitro studies to assess the antioxidative, radical scavenging and arginase inhibitory potentials of extracts from *Artocarpus altilis*, *Ficus exasperate* and *Kigelia africana*. Asian Pac. J. Trop. Biomed..

[31] Cooke M.S., Olinski R., Evans M.D. (2006). Does measurement of oxidative damage to DNA have clinical significance?. Clin. Chim. Acta.

[32] Valko M., Leibfritz D., Moncol J., Cronin M.T., Mazur M., Telser J. (2007). Free radicals and antioxidants in normal physiological functions and human disease. Int. J. Biochem. Cell Biol..

[33] Ismail N., Ismail M., Imam M.U., Azmi N.H., Fathy S.F., Foo J.B. (2014). Mechanistic basis for protection of differentiated SH-SY5Y cells by oryzanol-rich fraction against hydrogen peroxide-induced neurotoxicity. BMC Complement. Altern. Med..

[34] Smith R.A., Hartley R.C., Cocheme H.M., Murphy M.P. (2012). Mitochondrial pharmacology. Trends Pharmacol. Sci..

[35] Borner C. (2003). The Bcl-2 protein family: Sensors and checkpoints for life-or-death decisions. Mol. Immunol..

[36] Tomek M., Akiyama T., Dass C.R. (2012). Role of Bcl-2 in tumour cell survival and implications for pharmacotherapy. J. Pharm. Pharmacol..

[37] Hassa P.O., Hottiger M.O. (2008). The diverse biological roles of mammalian PARPS, a small but powerful family of poly-ADP-ribose polymerases. Front. Biosci..

[38] Sugawara T., Fujimura M., Noshita N., Kim G.W., Saito A., Hayashi T., Narasimhan P., Maier C.M., Chan P.H. (2004). Neuronal death/survival signaling pathways in cerebral ischemia. NeuroRx.

